# Explanatory Model of Dry Eye Disease Using Health and Nutrition Examinations: Machine Learning and Network-Based Factor Analysis From a National Survey

**DOI:** 10.2196/16153

**Published:** 2020-02-20

**Authors:** Sang Min Nam, Thomas A Peterson, Atul J Butte, Kyoung Yul Seo, Hyun Wook Han

**Affiliations:** 1 Department of Ophthalmology, CHA Bundang Medical Center, CHA University Seongnam Republic of Korea; 2 Bakar Computational Health Sciences Institute, University of California San Francisco San Francisco, CA United States; 3 Department of Ophthalmology, Institute of Vision Research, Eye and Ear Hospital, Severance Hospital, Yonsei University College of Medicine Seoul Republic of Korea; 4 Department of Biomedical Informatics, CHA University School of Medicine, CHA University Seongnam Republic of Korea

**Keywords:** dry eye disease, epidemiology, machine learning, systems analysis, patient-specific modeling

## Abstract

**Background:**

Dry eye disease (DED) is a complex disease of the ocular surface, and its associated factors are important for understanding and effectively treating DED.

**Objective:**

This study aimed to provide an integrative and personalized model of DED by making an explanatory model of DED using as many factors as possible from the Korea National Health and Nutrition Examination Survey (KNHANES) data.

**Methods:**

Using KNHANES data for 2012 (4391 sample cases), a point-based scoring system was created for ranking factors associated with DED and assessing patient-specific DED risk. First, decision trees and lasso were used to classify continuous factors and to select important factors, respectively. Next, a survey-weighted multiple logistic regression was trained using these factors, and points were assigned using the regression coefficients. Finally, network graphs of partial correlations between factors were utilized to study the interrelatedness of DED-associated factors.

**Results:**

The point-based model achieved an area under the curve of 0.70 (95% CI 0.61-0.78), and 13 of 78 factors considered were chosen. Important factors included sex (+9 points for women), corneal refractive surgery (+9 points), current depression (+7 points), cataract surgery (+7 points), stress (+6 points), age (54-66 years; +4 points), rhinitis (+4 points), lipid-lowering medication (+4 points), and intake of omega-3 (0.43%-0.65% kcal/day; −4 points). Among these, the age group 54 to 66 years had high centrality in the network, whereas omega-3 had low centrality.

**Conclusions:**

Integrative understanding of DED was possible using the machine learning–based model and network-based factor analysis. This method for finding important risk factors and identifying patient-specific risk could be applied to other multifactorial diseases.

## Introduction

### Background and Related Studies

Dry eye disease (DED) is defined as “a multifactorial disease of the ocular surface characterized by a loss of homeostasis of the tear film, and accompanied by ocular symptoms” [1]. Due to its multifactorial etiology, DED cannot be characterized by a single process and its management is complicated, in which finding the major causative factors behind DED is critical to appropriate treatment [1]. Therefore, identification of DED-related factors may enable advances in diagnosis, elucidative pathophysiology, therapy, and public education, as well as improvement of general and ocular health [2]. Indeed, various nonmodifiable, modifiable, environmental, and medical factors related to DED have been reported by observational studies and population-based, cross-sectional epidemiological studies [2]. DED risk factors are categorized as consistent, probable, and inconclusive; age, sex, Meibomian gland dysfunction (MGD), connective tissue disease, Sjogren syndrome, androgen deficiency, computer use, contact lens wear, estrogen replacement therapy, and medication use (eg, antihistamines, antidepressants, and anxiolytics) are identified as consistent risk factors [2].

Previously, a limited number of DED-associated factors were investigated using the Korea National Health and Nutrition Examination Survey (KNHANES) [3]. Although KNHANES consists of a large number of variables from health interview questionnaires, health examinations, and nutrition surveys, they were not fully utilized [3]. In addition, previous studies on DED have identified DED-related factors, instead of building a DED model to assess the risk of DED for new individuals [3-7].

### Highlights of This Study

In this study, we generated a point-based model with DED-associated factors from KNHANES using machine learning algorithms and Lasso regularization. These methods can improve the model performance to predict DED by selecting features from a large number of variables from a large dataset without overfitting while preserving complex interactions among features [8]. Furthermore, interactions among the factors were explored by network analysis. When the network analysis was applied to the model, a systemic understanding of DED, which cannot be achieved by conventional methods, was possible by showing the linkages between the relevant factors. To the best of our knowledge, this was the first attempt at building a machine learning–based model to evaluate the individual risks of DED and visualize the state using the network graph of DED-associated factors.

## Methods

### Overview of Survey Data

The design, methods, and data resource profile of KNHANES are available on the Web and in publications [9-11]. In short, KNHANES is an annual survey performed by the Korea Centers for Disease Control and Prevention (KCDC) in the Republic of Korea, which assesses the health and nutritional status of the population [10]. KNHANES is a nationwide cross-sectional survey of a representative set of 10,000 noninstitutionalized civilian individuals who are aged 1 year and older. Both DED assessment and food frequency surveys were conducted only in 2012. In the 2012 KNHANES, 192 primary sampling units (PSUs) were drawn from about 200,000 geographically defined PSUs nationwide; 20 final target households were sampled for each PSU as secondary sampling units [9]. KNHANES V (2012) was approved by the KCDC Research Ethics Committee (2012-01EXP-01-2C), and written informed consent was obtained from all subjects.

### Variable Inclusion

Four data files, HN12_ALL (health examination, health survey, and nutrient survey), HN12_ENT (ear, nose, and throat examination), HN12_EYE (eye examination), and HN12_FFQ (food frequency survey), were combined. DED was considered to be present when a subject had been diagnosed with DED by an ophthalmologist (the variable *E_DES_dg*) and was experiencing dryness (*E_DES_ds*). Conversely, patients were defined as DED-negative in the absence of both a diagnosis and symptoms. *E_DES_dg* and *E_DES_ds* are available for persons who are aged 19 years and older [11].

**Figure 1:**
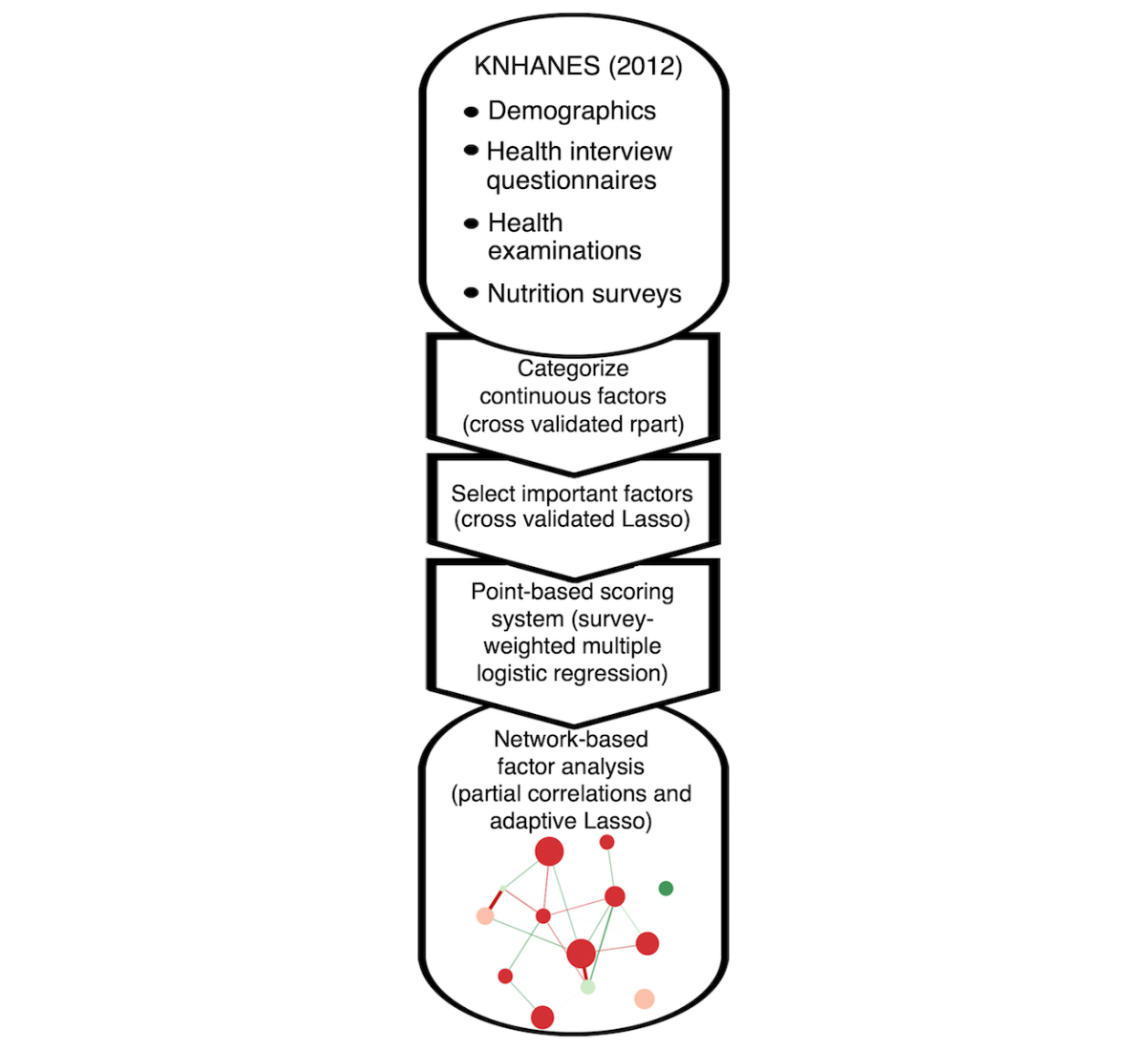
Flowchart of overall analysis steps. KNHANES: Korea National Health and Nutrition Examination Survey.

The included variables are listed in Textbox 1, and the overall analysis is summarized in Figure 1.

All variables were available for subjects aged 19 years and older except those of food frequency (19-64 years) and osteoarthritis radiology (≥50 years) [9]. The LDL level was calculated using the Friedewald equation, LDL=total cholesterol−(HDL+TG/5), with exclusion of TG levels of higher than 400 mg/dL [12].

**Included study variables of the Korea National Health and Nutrition Examination Survey data (2012).**

**Health examination data**

Physical examination

- Body mass index (BMI), rhinitis, sinusitis, blepharoptosis [11], and cataract

Blood test results

- Anemia, hemoglobin, hematocrit, iron, total iron-binding capacity, ferritin, hemoglobin A_1c_, white blood cell count, platelet count, red blood cell count, aspartate aminotransferase, alanine aminotransferase, creatinine, urea nitrogen, and vitamin D

Fasting (≥8 hours) blood parameters

- Sugar level, low-density lipoprotein cholesterol (LDL) level, high-density lipoprotein cholesterol (HDL) level, triglyceride (TG) level

Hypercholesterolemia definition: total cholesterol (TC)≥240 mg/dL or lipid-lowering medication

Hypertension definition: systolic blood pressure≥140 mm Hg, or diastolic blood pressure ≥ 90 mm Hg, or medication

Diabetes mellitus definition: fasting blood sugar level≥126 mg/dL, or diagnosis, or medication, or insulin injection

Fundus photography

- age-related macular degeneration, diabetic retinopathy

Osteoarthritis on radiology

**Health survey data**

- Age, educational stage, occupational class, household income, weight changes in the last year, mean duration of sleep per day, stress recognition, current smoker, frequency of drinking alcohol, activity level, lipid-lowering medications, diagnosed glaucoma, eye surgery, and menstruation

Diagnosed current diseases

- dyslipidemia, depression, stroke, myocardial infarction or angina, rheumatoid arthritis, thyroid disease, atopic dermatitis, and asthma

Diagnosed cancers

- stomach, colon, breast, cervix, and thyroid

**Food frequency survey data (daily intake)**

- Energy, carbohydrate, protein, total fat, n-3 polyunsaturated fatty acid, n-6 polyunsaturated fatty acid, saturated fatty acid, cholesterol, fiber, vitamin A, vitamin B1, vitamin B2, vitamin C, niacin, iron, calcium, potassium, phosphorus, and sodium

### Subsampling of Training and Test Sets and Categorization of Factors

Most DED cases (80.00%, 3513/4391) were used as training, and the other cases were used for testing. Likewise, non-DED cases were subsampled into training and test sets in the same way. Next, the categorization or recategorization of the factors was performed for the training set in consideration of reference values. Here, optimal cutoffs were determined by training a decision tree on the training data and using binarized decision tree rules as factors in the final regression model [13,14]. Missing values of each variable were classified as a separate class.

### Factor Selection Using Lasso (Least Absolute Shrinkage and Selection Operator)

Factors were transformed into dummy-coded variables, in which the largest category was used as reference and was excluded during model construction, and missing values were not included in the Lasso procedure.

Lasso trained using cross validation was applied to the transformed dummy variables with area under the curve (AUC) as a stopping metric and *wt_tot* as the sample weight for the analysis of the associations between the health interview, health examination, and nutrition survey. To regularize the model, we selected the optimal lambda using cross validation (*lambda.1se* in glmnet, ie, the lambda that yields an error one standard error away from the minimum error).

### Construction of a Model for Dry Eye Disease

Using the lasso-selected factors, a survey-weighted multiple logistic regression model was constructed from the complex survey design of KNHANES. The survey design was represented using the variable *psu* for PSU and *ID_fam* for the secondary sampling unit, *kstrata* for strata, and *wt_tot* for weights.

### Developing a Point-Based Scoring System for Dry Eye Disease

A point-based scoring system was developed by multiplying the coefficients of factors in the survey-weighted regression model by 10 and rounding to the nearest integer [15]. The total score of each individual in the training set was determined by summing the points for factors accurately describing that individual. Next, performance was assessed using weighted receiver-operating characteristic (ROC) curves and the AUCs with survey sample weight (*wt_tot*). An optimal cutoff for the point-based system was determined by maximizing Youden’s index value (sensitivity+specificity−1).

### Testing the Point-Based Scoring System for Dry Eye Disease

The model’s performance was assessed using the test set. The AUC’s confidence interval was calculated; sensitivity and specificity were reported using the point-based system’s cutoff determined from the training set.

### Analysis of Dry Eye Disease-Risk Factors

A survey-weighted multiple logistic regression analysis was performed using the factors selected by lasso. Odds ratios (ORs) were calculated by exponentiating the coefficient derived by logistic regression. Estimated population counts and proportions for categories were computed.

### Network Analysis of Dry Eye Disease-Associated Factors

With the training set, a correlation matrix for the DED-associated factors was created. Weighted Pearson correlation coefficients between two variables were calculated. Next, a network graph was plotted by setting the graph argument to “glasso” and the layout to “spring.” A partial correlation network was drawn using the graphical lasso algorithm and the Extended Bayesian Information Criterion by which false positive edges were controlled. Each edge represents the relationship between 2 nodes after controlling for all other relationships in the network [16,17]. The Fruchterman-Reingold algorithm is applied with the “spring” layout, in which the lengths of edges are dependent on their absolute weights [16]. Green edges indicate positive weights (correlations) and red edges indicate negative weights. Color saturation and edge width correspond to the absolute weight relative to the strongest weight in the graph. Node size was proportional to the *z*-score for the absolute point of the factor. Nodes were grouped as *significant* (*P*<.05, risk factor analysis) or *possible* (*P*≥.05, risk factor analysis).

Three centrality indices (strength, closeness, and betweenness) were computed. Centrality is the absolute sum of the edge weights connected to the node, closeness is the sum of the shortest distances from the node to all other nodes in the network, and betweenness is the number of times in which the node lies on the shortest path between 2 other nodes [17,18].

### Statistics and Software

R version 3.6.1 and variable functions from its packages were used: decision tree, “rpart” in the caret package (using down-sampling and cross-validation) [19,20]; dummy-coded variables, “dummy.code” in the psych package [21]; cross-validation for Lasso, “cv.glmnet” in the glmnet package [22]; survey-weighted multiple logistic regression, “svyglm” in the survey package [23]; weighted ROC curve and AUC, “WeightedROC” and “WeightedAUC,” respectively, in the WeightedROC package [24]; confidence interval of AUC, “withReplicates” in the survey package [23]; estimation of population counts and proportions, the survey package [23]; general graphs, the ggplot2 package [25]; weighted correlation, “wtd.cor” in the weights package [26]; network graph, “qgraph” in the qgraph package [16]; and centrality indices, “qgraph” and “centralityTable” in the qgraph package [16].

## Results

### Point-Based Scoring Model for Dry Eye Disease

Total sample sizes for DED and non-DED were 575 and 3816 cases, respectively. The estimated prevalence of DED was 10.5% (SE 1.0%): 5.3% (SE 1.0%) for men and 15.9% for women (SE 1.0%). A total of 13 factors were selected by lasso and the point-based scoring system for each factor is outlined in Table 1.

**Figure 2:**
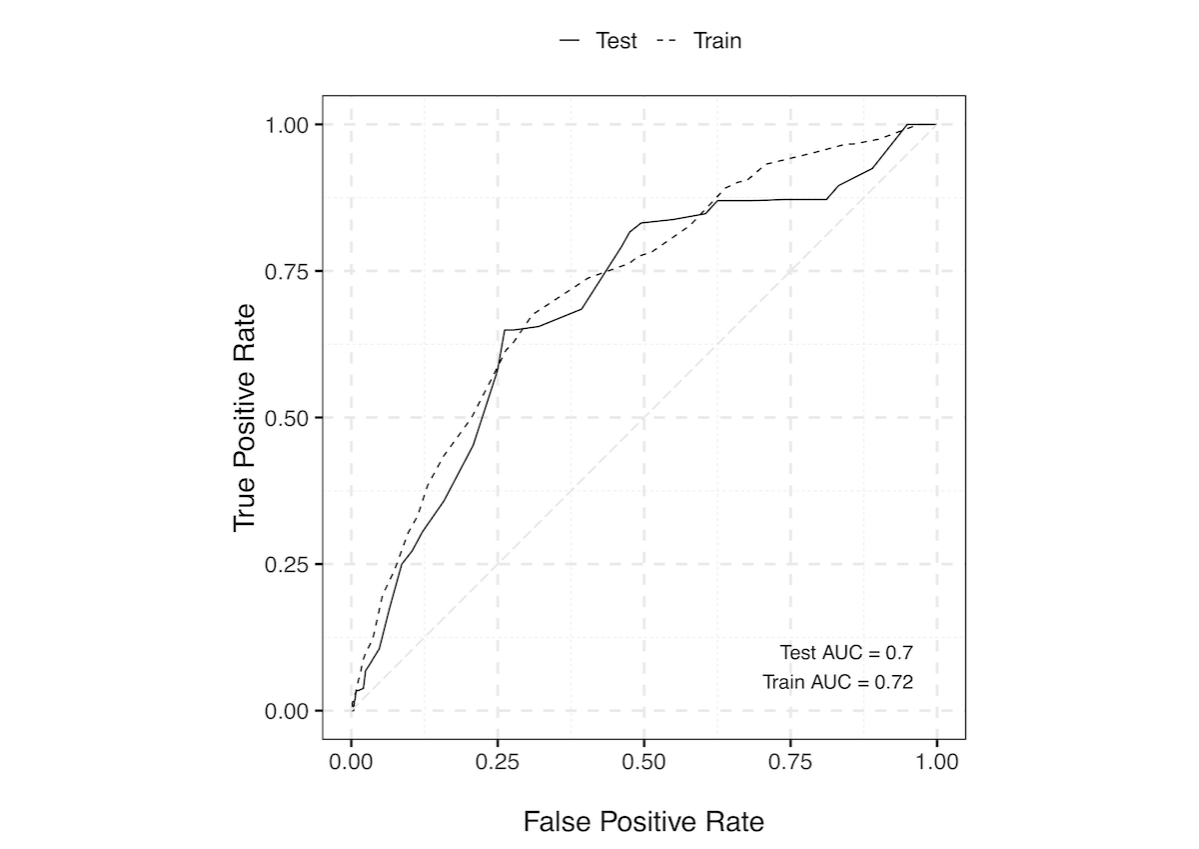
Weighted receiver-operating characteristic (ROC) of the point-based scoring system for predicting dry eye disease. ROCs for train and test sets were compared. AUC: area under the curve.

Using this scoring system on the test set achieved an AUC of 0.70 (95% CI 0.61-0.78; Figure 2). Sensitivity and the specificity were 0.66 and 0.68, respectively, at a cutoff of 10 points.

**Table 1:** Point-based scoring system for assessing individual risk of dry eye disease using coefficients from a survey-weighted multiple logistic regression model.

Variables	Regression coefficient (beta)	Standard error	Points^a^
Women	.865	0.182	9
Corneal refractive surgery	.903	0.281	9
Current depression	.709	0.294	7
Eye surgery: cataract	.705	0.196	7
Perceived stress: much to extreme	.560	0.136	6
Other ocular surgeries	.646	0.258	6
Phosphorus intake <746 mg/day	.454	0.182	5
Age 54-66 years	.396	0.179	4
Rhinitis by physical examination	.384	0.144	4
Lipid-lowering medications	.408	0.194	4
Cholesterol intake ≥240 mg/day	−.145	0.151	−1
Current smoker	−.441	0.242	−4
Omega-3 intake, 0.43%-0.65% kcal/day	−.407	0.172	−4

^a^Calculated by multiplying the coefficient of the variable by 10 and rounding to the nearest integer. A positive point means a positive predictor for dry eye disease. Dry eye disease is indicated when the sum of all points is 10 or higher.

### Risk Factor Analysis For Dry Eye Disease

In the risk factor analysis, 10 of the 13 variables were significant (*P*<.05; Table 2). The top 3 significant risk factors in the point-based model were women, corneal refractive surgery, and current depression (Tables 1 and 2). Omega-3 intake between 0.43% (1003 mg for total 2100 kcal) and 0.65% (1517 mg for total 2100 kcal) was a significant protective factor.

Population counts (n), proportions (%), and ORs were estimated according to complex survey design. ORs and *P* values were calculated by multiple logistic regression including all listed variables. The missing data category for each variable were included for calculation but not shown in the table.

**Table 2:** Population counts (n), proportions (%), and odds ratios of variables in the points-based scoring system for dry eye disease.

Factors		Healthy, n (%)		Dry eye disease, n (%)		OR^a^ (95% CI)		*P* value	
**Sex/gender**									
	Men		16,277,579 (54.19)		911,587 (25.90)		Reference		Reference
	Women		13,761,300 (45.81)		2,607,754 (74.10)		2.6 (1.8-3.6)		<.001
**Perceived stress**									
	None to a little		21,872,165 (72.81)		2,177,913 (61.88)		Reference		Reference
	Much to extreme		6,864,096 (22.85)		1,202,940 (34.18)		1.6 (1.2-2.0)		<.001
**Eye surgery**									
	None		26,673,187 (88.80)		2,701,904 (76.77)		Reference		Reference
	Cataract		1,185,785 (3.95)		276,588 (7.86)		1.8 (1.2-2.6)		.004
	Corneal refractive		1,150,662 (3.83)		339,857 (9.66)		2.8 (1.7-4.6)		<.001
	Other		1,029,243 (3.43)		200,991 (5.71)		1.6 (1.0-2.5)		.08
**Rhinitis on inspection**									
	No		21,295,695 (70.89)		2,180,596 (61.96)		Reference		Reference
	Yes		8,047,298 (26.79)		1,243,634 (35.34)		1.5 (1.2-2.0)		.001
**Lipid-lowering medications**									
	No		27,360,794 (91.08)		3,087,286 (87.72)		Reference		Reference
	Yes		1,315,445 (4.38)		282,072 (8.01)		1.5 (1.1- 2.0)		.02
**Age (year)**									
	<54 or ≥66		25,121,235 (83.63)		2,757,338 (78.35)		Reference		Reference
	54-66		4,917,644 (16.37)		762,003 (21.65)		1.4 (1.1-2.0)		.02
**Current depression**									
	No		25,591,681 (85.20)		2,616,142 (74.34)		Reference		Reference
	Yes		456,573 (1.52)		153,162 (4.35)		1.9 (1.1-3.3)		.02
**Omega-3 intake (kcal/day)**									
	<0.43% or >0.65%		13,804,921 (45.96)		1,812,313 (51.50)		Reference		Reference
	0.43%-0.65%		10,414,321 (34.67)		857,985 (24.38)		0.7 (0.5-1.0)		.04
**Phosphorus intake (mg/day)**									
	≥746		19,948,841 (66.41)		1,940,991 (55.15)		Reference		Reference
	<746		4,270,401 (14.22)		729,307 (20.72)		1.4 (1.0-2.0)		.09
**Current smoker**									
	No		21,334,370 (71.02)		2,950,840 (83.85)		Reference		Reference
	Yes		7,399,648 (24.63)		430,013 (12.22)		0.7 (0.5-1.1)		.14
**Cholesterol intake (mg/day)**									
	<240		12,037,627 (40.07)		1,575,001 (44.75)		Reference		Reference
	≥240		12,181,614 (40.55)		1,095,296 (31.12)		0.9 (0.7-1.2)		.57

^a^OR: odds ratio.

### Network Analysis for Dry Eye Disease Model

**Figure 3:**
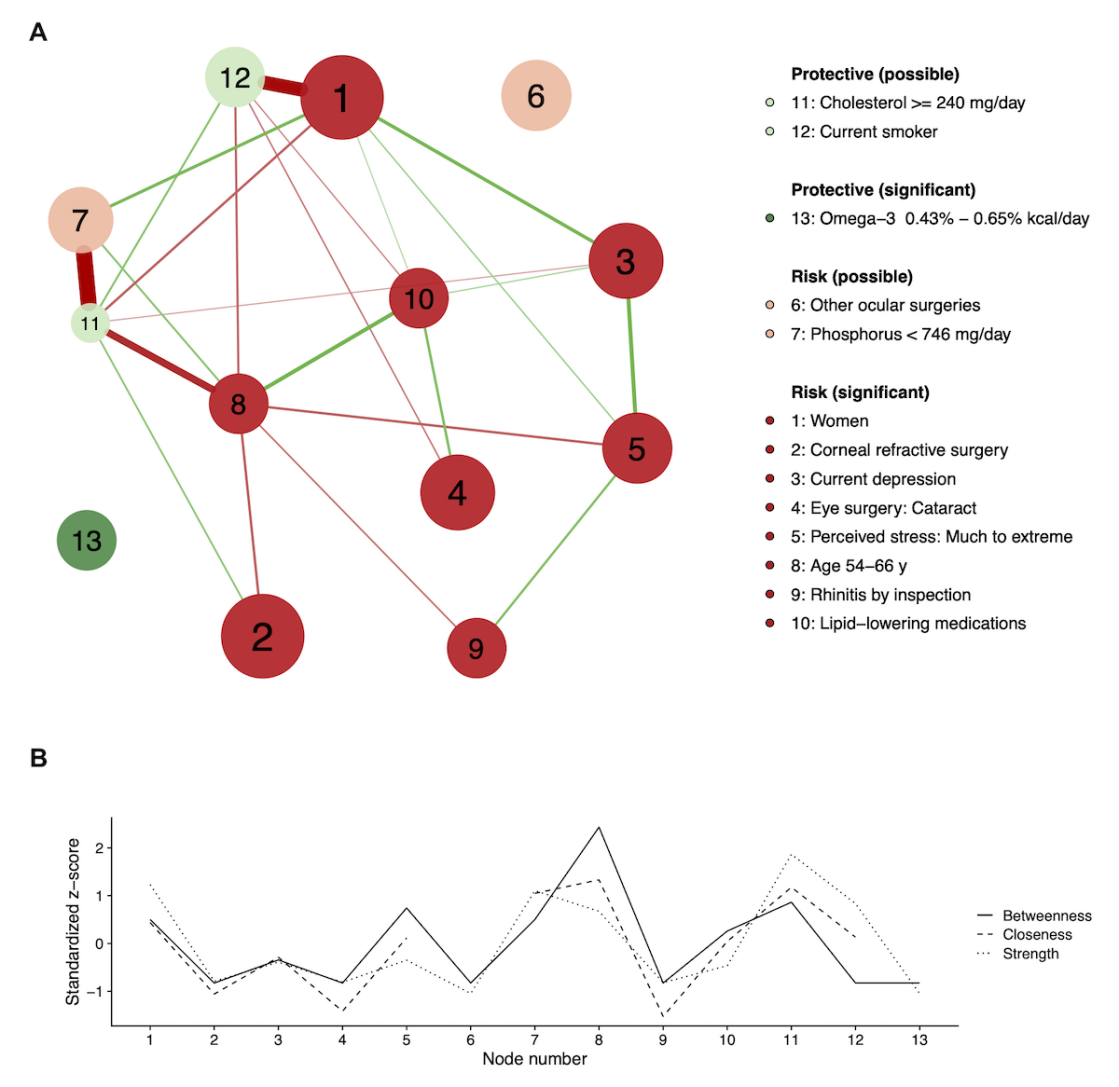
The partial correlation (adaptive LASSO) network (A) and the centrality indices (betweenness, closeness, and strength) (B) of the factors associated with the dry eye disease. Factors were positively (risk) or negatively (protective) associated with dry eye disease. Factors can be significant (*P*<.05) or possible (P≥.05) according to the risk factor analysis. The node size is proportional to the absolute value of the point for the node’s variable. Green and red edges mean positive and negative connections, respectively. The edge with the highest absolute weight will have full color saturation and be the widest.

In Figure 3, model factors are depicted in a partial correlation network with centrality indices. The network-based factor analysis in Figure 3 allows for the interrogation of the interrelatedness of factors associated with DED, with larger nodes representing factors’ importance (points), green nodes representing protective factors, and red nodes representing risk factors. According to centrality indices (Figure 3), *Age 54-66y* (node 8) had high centrality in the network. For *other ocular surgeries* (node 6) and *omega-3* (node 13), the closeness indices were too low to calculate owing to lack of the connections to other nodes.

Four significant risk factors were linked in succession from *women* (node 1) to *current depression* (node 3), *much to extreme stress* (node 5), and *rhinitis* (node 9). Other serial connections were found in three significant risk factors, *age 54-66y*, *lipid-lowering medication* (node 10), and *cataract surgery* (node 4). Nonsignificant factors were strongly connected with other significant factors, for example, *current smoker* (node 12) to *women*, and *cholesterol intake* (node 11) to *age 54-66y*. Another nonsignificant factor, *phosphorous intake* (node 7), was closely associated with the *cholesterol intake*.

## Discussion

### Principal Findings

Our model showed moderate performance for DED prediction with a point-based scoring system in which the maximum AUC might reach 0.78. Our study chose a stricter definition of DED because the individuals were not only required to be symptomatic but also have a physician diagnosis. In addition, the absence of DED was rigorously defined as a lack of symptoms and no physician diagnosis in the past. According to the TFOS DEWS II report, DED is diagnosed on the basis of the presence of a symptom and positivity for one or more homeostatic markers [1]. Our DED definition more reflected a diagnosis of DED, and thus, the prevalence could be lower than that of prior studies that used a symptomatic definition [2]. However, even our definition was imperfect because diagnostic tests were not performed and might be biased by the availability of a clinic in the local area or by the respondent’s condition. This may explain, in part, the moderate diagnostic performance of our DED model.

### Reasoning for Machine Learning and Point-Based Scoring Model

Machine learning algorithms and techniques were used for several purposes. First, tree-based machine learning was applied to categorize continuous variables. Second, Lasso was implemented to select important factors to simplify the model and to reduce overfitting. Third, the models were generated using a training sample and validated with a separate test sample, which enabled estimation of predictive power. This technique is preferred because standard regression modeling and automated variable selection (eg, stepwise selection, pretesting of candidate predictors) can result in overfitting [27,28]. As a result, our model was robust enough to generalize to populations not used during training without overfitting (Figure 2).

Point-based scoring systems are useful for describing the relationship between multiple factors and the risk of the development of a disease [15]. Likewise, using our point-based model, DED can be assessed by summing the points accurately describing an individual with a cutoff of 10 points, indicating high risk for DED. In addition, the node size was determined by its point, and interrelatedness of DED risk factors was interrogated. Because DED was predicted by the sum of points, larger nodes might be prioritized in evaluating DED.

### Interpretation for Indirect Model Factors With Network Analysis

By risk factor and network analyses, significant factors were presumed to be directedly associated with DED, whereas nonsignificant factors might be indirectly associated. Conventionally, nonsignificant factors might have been confounding variables that are related to DED via other significant factors. The network graph showed that nonsignificant factors such as *phosphorus <746 mg/day* (node 7), *current smoker* (node 12), and *cholesterol ≥240 mg/day* (node 11) were connected to significant factors such as *women* (node 1) and *age 54-66y* (node 8; Figure 3). However, those nonsignificant factors were necessary to maximize the model performance and selected by a machine learning–based Lasso regression. Therefore, they seemed to be included to tune points of other significant factors without a causal effect on DED. For example, *current smoker* (node 12) had a negative effect on node 1 (*women*) because it generally occurred in men rather than women. Smoking has been reported as an inconclusive risk factor for DED, and our study did not suggest smoking as a risk factor [2].

### Known Factors in Dry Eye Disease Model

In the network-based analysis in Figure 3, *age 54-66y* (node 8) showed high centrality in the network, which means that it has more connections (strength), it is closer to other nodes (closeness), or makes connections between other nodes (betweenness). This high-centrality node exists at the center of the network and acts as hubs that connect disparate nodes [18]. In contrast, *omega-3 intake* (node 13) and *other ocular surgeries* (node 6) were independent nodes with low centrality.

In the previous study with KNHANES 2010-2011 by Ahn et al [3], 50- to 59-year-old and 60- to 69-year-old groups are presented as risk factors, which are in agreement with our age factor of 54 to 66 years. Other risk factors suggested (women, extreme stress, cataract surgery, refractive surgery, other ocular surgery) were also picked up in our model except for thyroid disease and educational level [3]. Thyroid disease is a possible risk factor, and the previous study argues an ambiguous link between thyroid disease and DED [2,3]. The difference between our work and the previous study can come from different definitions of DED because we used both the diagnosis and symptoms to classify an individual as having DED, whereas the previous study used the criteria of having either the diagnosis or symptoms [3].

Female sex is consistently associated with DED throughout the studies, but the prevalence of DED is considerably variable in these studies with respect to sex and age [2]. Stress has been associated with DED as a trigger or an immune response modulator [2,3]. Ocular surgery can cause DED in various ways, for example, the exposure to strong light of the microscope during the surgery, use of anesthetic or postoperative eyedrops, and the corneal nerve damage [3]. Specifically, refractive surgery leads to neuropathic dry eye by sensory nerve damage, decreased tear secretion, and induced neurogenic inflammation [2].

### New Factors in Dry Eye Disease Model

Depression, rhinitis, lipid-lowering medication, and omega-3 intake were new DED-associated factors in our model that were added to previously reported factors of KNHASES [3]. Those factors have not been evaluated in the previous KNHASES study on DED [3]. Depression (node 3) was positively connected to node 1 (women), and a close association between depression and DED in women has been reported [7]. Depression is more prevalent in patients with DED partly because of somatization and perceptional changes in ocular discomfort [29]. In addition, depression was serially connected to other risk factors (Figure 3), such as female sex, stress, and rhinitis, which may be utilized for DED risk evaluation and control because positively connected serial factors can occur together with possible causalities. For rhinitis, allergic rhinitis was reported to be significantly associated with DED, and inflammation is related to both [30,31]. Notably, rhinitis was a clinically reliable factor because it was diagnosed by physician’s examination.

Other serial risk factors were *age 54-66y* (node 8), *lipid-lowering medication* (node 10), and cataract surgery (node 4). Dyslipidemia and its treatment might be an issue for the 54- to 66-year-old group, which could explain the negative connection between node 8 and node 11 (*cholesterol >240 mg/day*). Dyslipidemia has been suggested to induce MGD, a major cause of DED [5,32]. However, oral statin therapy, not hypercholesterolemia, were recently reported to be associated with the symptoms of DED [33]. Interestingly, sterols have been reported to reduce cataract severity [34,35], and cholesterol metabolism might be linked to cataract formation [36].

The results of randomized controlled trials for DED treatment effect of omega-3 have been inconsistent, and larger studies suggest no statistically significant improvement compared with placebo [37,38]. Nonetheless, omega-3 has been commonly used to treat DED in the clinic because essential fatty acids, including omega-3, display anti-inflammatory properties [39], enhance the lipid layer of the tear film, and improve tear secretion while lacking association with substantial side effects [2]. However, it remains a problem that there is no consensus on the dose of supplementation, and our study suggested that 1000 to 1500 mg daily intake of omega-3 (for 2100 kcal average calorie intake) helped to prevent DED. It was noteworthy that *omega-3 intake* might be used to treat DED without possible effects on other factors because it did not have a connection in the network (Figure 3).

### Limitations

This study has several limitations. Eye-related factors (blepharitis, lid abnormalities, low blink rate, other ocular surface disease, or conjunctivochalasis) and Sjögren syndrome could not be assessed [40]. In addition, some nutrient factors might have been missed because nutrient intake data were available only for subjects younger than 65 years [9].

### Conclusions

In summary, the machine learning–based model to assess the individual risks of DED was successfully created from a large-scale national survey data. With this model, additional DED-associated factors could be suggested, and personalized medical advice was possible using the network graph of the model factors. These approaches allowed integrative understanding of DED and may be applied to other multifactorial diseases.

## Abbreviations

AUC: area under the curve
DED: dry eye disease
KCDC: Korea Centers for Disease Control and Prevention
KNHANES: Korea National Health and Nutrition Examination Survey
MGD: Meibomian gland dysfunction
NRF: National Research Foundation of Korea
OR: odds ratio
ROC: receiver-operating characteristic
